# A genome-wide transcriptional activity survey of rice transposable element-related genes

**DOI:** 10.1186/gb-2007-8-2-r28

**Published:** 2007-02-27

**Authors:** Yuling Jiao, Xing Wang Deng

**Affiliations:** 1 Department of Molecular, Cellular and Developmental Biology, Yale University, 165 Prospect Street, New Haven, CT 06520, USA

## Abstract

**Background:**

Transposable element (TE)-related genes comprise a significant portion of the gene catalog of grasses, although their functions are insufficiently characterized. The recent availability of TE-related gene annotation from the complete genome sequence of rice (*Oryza sativa*) has created an opportunity to conduct a comprehensive evaluation of the transcriptional activities of these potentially mobile elements and their related genes.

**Results:**

We conducted a genome-wide survey of the transcriptional activity of TE-related genes associated with 15 developmental stages and stress conditions. This dataset was obtained using a microarray encompassing 2,191 unique TE-related rice genes, which were represented by oligonucleotide probes that were free from cross-hybridization. We found that TE-related genes exhibit much lower transcriptional activities than do non-TE-related genes, although representative transcripts were detected from all superfamilies of both type I and II TE-related genes. The strongest transcriptional activities were detected in TE-related genes from among the MULE and CACTA superfamilies. Phylogenetic analyses suggest that domesticated TE-related genes tend to form clades with active transcription. In addition, chromatin-level regulations through histone and DNA modifications, as well as enrichment of certain *cis *elements in the promoters, appear to contribute to the transcriptional activation of representative TE-related genes.

**Conclusion:**

Our findings reveal clear, albeit low, general transcription of TE-related genes. In combination with phylogenetic analysis, transcriptional analysis has the potential to lead to the identification of domesticated TEs with adapted host functions.

## Abstract 2

A genome-wide survey of the transcriptional activity of TE-related genes that were associated with fifteen developmental stages and stress conditions revealed clear, albeit low, general transcription of TE-related genes.

## Background

The completion of the rice (*Oryza sativa*) genome sequence allowed further functional classification of the coding sequences of this important crop and model of grass species [1,2]. Detailed annotation of the rice genome revealed that nearly a quarter of the rice open reading frame (ORF) coding capacity has features of transposable elements (TEs) and are therefore defined as TE-related genes [3]. Like other genes, these TE-related genes have predicted normal gene structure with protein coding capacity. However, they share significant sequence similarity with known TEs in either or both of the following ways: they have TE signature sequences in The Institute for Genomic Research (TIGR) *Oryza *Repeat Database [4] or they contain TE-related protein domains [3]. By this definition, TE-related genes can include potentially active TEs (based on the existence of a functional ORF) as well as cellular genes derived from TEs. Many of these TE-related genes encode reverse transcriptases, transposases, or other related proteins [5], and they can be further classified based on protein domain and other sequence features [3,4]. Those TEs overwhelming in number that lack functional ORFs are not considered to be genes [3]. Although there are many TE-related genes, the biologic functions of these genes remain elusive [6].

TEs are considered to be important for the maintenance and diversification of genomes. TEs are usually separated into two classes that differ in the mode of propagation: retrotransposons, or type I elements, which transpose by reverse transcription of an RNA intermediate; and type II elements, which only use a DNA intermediate in movement within the genome. Both classes can be further divided into several superfamilies, each with a unique evolutionary history. Representatives of virtually all superfamilies of TEs have been detected in grass genomes [7-9]. Accumulating evidence suggests that TE activities have profound impact on the genome [5], influencing genome size, genome rearrangement, chromatin transcription, and gene evolution [10-15]; many of these factors relying specifically on the transposition activity of TEs.

Although most TEs are considered inactive [16,17], there have been isolated reports of TE transposition in rice and other grasses [18]. A common condition promoting transposition is stress, including that which occurs in *in vitro *cell or tissue culture [19-22]. Developmental regulation of transposition has also been reported in intact plants [23,24].

Transcription of TE-related genes is required for their own transposition and that of other related TEs, although transcription itself may not be sufficient for transposition [20,25,26]. Analysis of TE-related genes from certain subgroups of the type I class and the *Mutator*-like superfamily of the type II class suggests that their transcripts are widely present in grasses [27,28]. Most of these transcribed TEs have coding capacity and are therefore considered TE-related genes. A recent study of expressed sequence tags (ESTs) in sugarcane identified 267 active TE-related transcripts [29]. Transcription of TE-related genes was also reported in an unbiased survey of the transcriptional activity of a single rice chromosome using a tiling microarray [30].

Apart from the potentially active TEs among these TE-related genes, domesticated TE-related genes, which acquire new functions for the host, have also been found to exist. Although our current classification for distinguishing TE-related genes from non-TE-related genes is not definitive [31], two recent studies in *Arabidopsis *identified domesticated TE-related genes contributing to cellular processes [32,33]. Similar examples were also found in animals [34,35]. Such findings in part support the hypothesis that TE-related genes may influence the evolution of their host by providing a source of novel coding capacity.

The potential impact of domesticated TE-related genes on the evolution of genomes requires systematic investigation. One attempt to identify further domesticated TE-related genes is sequence mining [36]. Because a change of position through transcription can be detrimental to the host, transposon-derived genes with known host function usually lack mobility. As a consequence, they may be devoid of transposon-specific terminal sequences [32,36]. By employing this criterion in a search, one particular member of the MULE superfamily was identified as a domesticated gene candidate [36]. Transcription is an important feature of domesticated TE-related genes, because it is generally required in cellular functions of the host [32,33]. By surveying transcriptional activity and combining other approaches, we would be able to identify domesticated TE-derived gene candidates.

Another mechanism for the evolution of new genes from TEs is through their ability to acquire and fuse fragments of genes to new genomic locations, as seen in plant Pack-MULE and, more recently, in certain *Helitron*-like and CACTA elements [13,14,37,38]. However, many of these Pack-MULEs have been suggested to possess pseudogene-like features [39]. Pack-MULE, as a unique group of TE-related genes, is relatively well annotated and is a current focus of interest regarding the origin of genes [37].

Given the paucity of information on TE-related genes, a systematic study of their transcriptional activity in a well characterized genome is required to enhance our understanding of the activity of TE-related genes. That the sequence of the rice genome is now completely annotated makes it a good resource for such a genome-wide survey [3]. Recent advances in microarray technology allow us to study the transcriptional activity of genes in a high-throughput manner. It is therefore possible to conduct a genome-wide survey of the transcriptional activity of rice TE-related genes, especially those more divergent ones for which unique oligomer probes can be designed. Different from simple TEs composing mostly repetitive sequences, many TE-related genes are diverged enough to have short oligomers representing their unique sequence regions. Such an approach has recently been utilized to analyze transcription of TE-related genes in plants and animals [11,30,40]. In addition to TE-related genes, TEs without protein-coding capacity and other tandem repeats may also exhibit transcriptional activity [26,41]. Transcripts derived from tandem repeats in the heterochromatin can give rise to small RNAs, which in turn direct the modification of histones and DNA in TE-related sequences and nearby regions by means of RNA interference [16]. Although transcripts from tandem repeats are important for the genome, their highly repetitive nature prohibits characterization of their unique identities in chromosomal organization on a genome-wide scale [42,43].

We conducted an expression analysis for rice TE-related genes using 70-mer oligonucleotide microarrays. Expression profiles from 4,728 oligonucleotides covering organs from rice plants were analyzed under both normal conditions at various developmental stages as well as under stress conditions. Clear but restricted transcription of TE-related genes were found for all major superfamilies of TE-related genes. Mechanisms controlling representative TE transcription were further analyzed.

## Results

### Representation of TE-related genes by an oligonucleotide microarray

A 70-mer oligonucleotide set was previously developed to span the rice genome [44]. Many TE-related genes are included in this oligomer set design, allowing survey of a large number of rice TE-related genes. However, for the sake of simplicity, those oligonucleotide probes representing TE-related genes were removed from analysis in all prior genome profiling analyses [44-47]. Here, we collected all of our available datasets and systematically examined the transcriptional activities of TE-related genes in various tissues and growth conditions. In particular, we included datasets representing cell cultures and stress-exposed tissues.

According to the rice genome annotation at TIGR [3] and a literature review [27,48], a total of 14,404 genes were identified as TE-related genes, based on the presence of TE signature sequences in the TIGR *Oryza *Repeat Database [4] or TE-related Pfam domains. Among these TE-related genes, 9,493 were classified as type I (retrotransposons) TE-related genes and 4,159 were classified as type II (DNA transposon) TE-related genes. These TE-related genes were further classified into superfamilies according to sequence signatures (Table 1). The classification at TIGR was followed, modified in accordance with recently published studies [27,48]. There were another 752 TE-related genes without further classification. A remapping of oligonucleotides in our microarray [44] to annotated genes indicated that 2,191 (15.2%) TE-related genes were represented by at least one 70-mer oligonucleotide that was free from cross-hybridization (see Materials and methods, below). Most oligomers, if not all, mapped to unique coding regions instead of repetitive sequences. In addition, 1,966 70-mer oligonucleotides mapped to more than one TE-related gene while remaining cross-hybridization free from non-TE-related genes. These oligonucleotides covered another 9,396 (65.2%) TE-related genes.

### Transcriptional activity of TE-related genes

To obtain a comprehensive picture of the transcriptional activity of TE-related genes, we assembled their transcription profiles into a collection of 15 datasets acquired from various tissues and under various physical conditions (Table 2). Five tissues grown under normal conditions from different developmental stages, four cell cultures, and six tissue samples under conditions of salinity or drought were included [44-47]. Three or more independent biologic replicates for each sample were analyzed. In order to assemble a compendium of transcription profiles with minimal sample variation, quantified microarray hybridization signals from different experiments were pulled together and subjected to an automatic processing pipeline, with manual inspection to correct for slide background, normalize experimental variations, filter problem spots, and check data quality. A previously described method, which takes into account both negative and positive controls as well as data reproducibility, was applied here to determine the expression threshold [44]. Such an experimental expression threshold was also supported by reverse transcription (RT)-polymerase chain reaction (PCR) of randomly selected genes.

**Figure 1:**
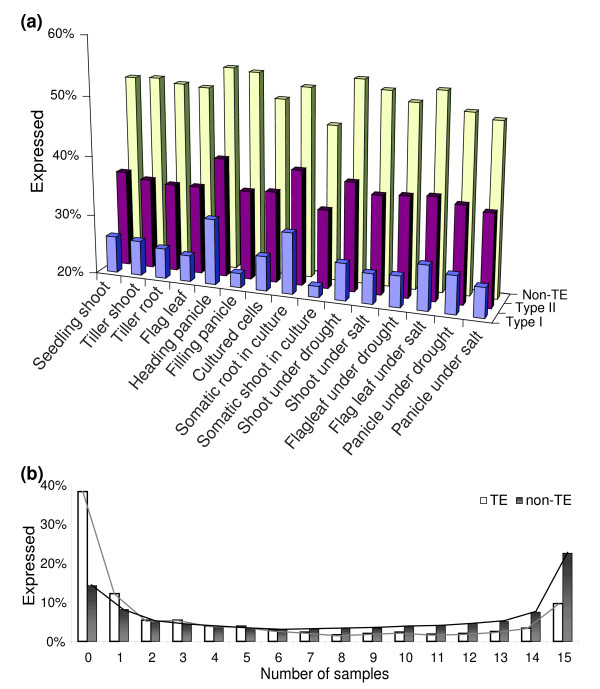
Summary of expression of TE-related genes. **(a) **Percentage of the transcribed type I and type II TE-related genes and non-TE-related genes in different samples. Percentages of transcribed genes in each category are shown for all samples. **(b) **Levels of transcription can be inferred based on how often (in how many different samples) expression was detected for TE-related and non-TE-related genes. TE, transposable element.

Examination of the expression of TE-related genes in each sample indicates that heading stage panicle has the greatest level of detected expression at 33%, whereas expression percentage in somatic shoot culture is the lowest, at 26% (Figure 1a). We also found that DNA transposons (type II) have 11% to 18% higher expression percentage than retrotransposons (type I) in all samples analyzed (Figure 1a).

By monitoring the expression of 2,191 TE-related genes using unique oligomer probes, we identified expression of 1,084 (61.7%) TE-related genes in at least one of our 15 samples. This is in contrast to findings in non-TE-related genes, 85.8% of which are expressed in at least one sample and 22.6% in all samples, using the same selection criteria. Expressed TE-related genes tend to exhibit transcription in a relatively small number of samples. The percentages of expressed TE-related genes in a wide range of samples are markedly lower than those of non-TE-related genes (Figure 1b). For those oligonucleotide probes that match multiple TE-related genes, 73.7% and 5.1% had hybridization signals in at least one sample or in all samples, respectively. Considering that those probes match multiple repetitive genes, a smaller portion of those TE-related genes that they represent is expected to be transcribed.

**Figure 2:**
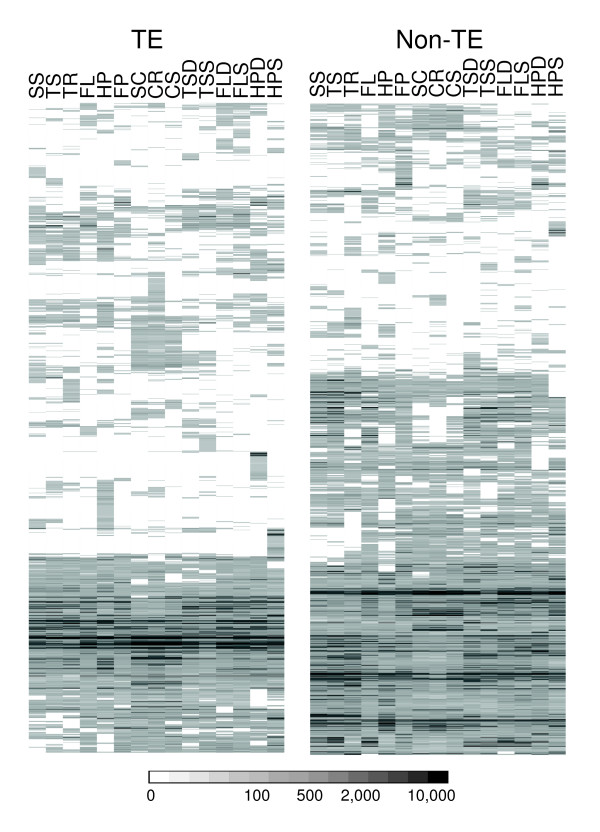
Global expression map showing transcriptional activity of TE-related and randomly selected non-TE-related genes. Only 1,353 TE-related genes with transcription in at least one sample are included. Another 1,353 non-TE-related genes randomly picked from those with transcription in at least one samples are shown in parallel. Each lane represents one sample in the same order as in Table 2. Shades of gray indicate the magnitude of transcription signals, which are based on microarray hybridization signals without units. TE, transposable element.

To probe quantitatively for the transcriptional activity of TE-related genes, the expression intensities of those 1,084 transcribed TE-related genes and an similar number of randomly selected transcribed non-TE-related genes are visually juxtaposed after clustering (Figure 2). Even though only transcribed genes are being compared here, it is clear that the transcription of TE-related genes was in general weaker than that of their non-TE-related counterparts. Furthermore, a large portion of the transcribed TE-related genes exhibited detectable transcription in fewer rice samples than was the case for non-TE-related genes. However, there are clearly a few clusters of TE-related genes with rampant transcription in most rice samples, and some of this transcription is quite marked (Figure 2). A few organ-specific clusters, such as one for cultured cells (lanes 7, 8 and 9 in Figure 2), were also found.

To gauge the reliability of our microarray data for TE-related genes, we first compared rice cDNA and EST collections with our data. We found 496 TE-related genes in the cDNA/EST collection in TIGR database [3]. These cDNAs and ESTs were derived from six rice samples: callus, seed, shoot and stem, leaf, root, and flower (heading panicle). We have similar (although not identical) rice samples with microarray expression profiles for all of them except seed. A survey of these TE-related cDNAs/ESTs indicates that 80% of those covered by our microarray also had detectable transcription. We further used RT-PCR to verify the microarray data. An attempt to amplify a series of TE-related genes with different levels of microarray signals supported our choice of threshold used to determine expression. Of the 10 genes with expression level within 100 units above the threshold, seven were amplified by RT-PCR; in contrast, only two out of 10 with expression below the threshold were amplified. Moreover, 34 randomly selected TE-related genes identified through microarray analysis as being shoot expressed were tested with RT-PCR using seedling shoot RNA samples. Twenty-nine (85%) of them were clearly detected. An independent tiling microarray analysis of rice transcriptome also covered a significant portion of the TE-related genes [43]. A preliminary survey of the transcriptional activities of TE-related genes in this dataset gives a similar portion of expression (about 30%) among tissues examined [49], although a different platform and hybridization detection procedure were used [43].

### Transcription of type I TE-related genes

In addition to taking an inventory of transcribed TE-related genes in various tissues and under multiple growth conditions, the availability of high-quality complete genome sequence provided an opportunity to elucidate how transcriptional activities evolve following sequence divergence. To this end, phylogenic trees were generated for all major TE-related gene superfamilies and were integrated with their members' expression profiles.

**Figure 3:**
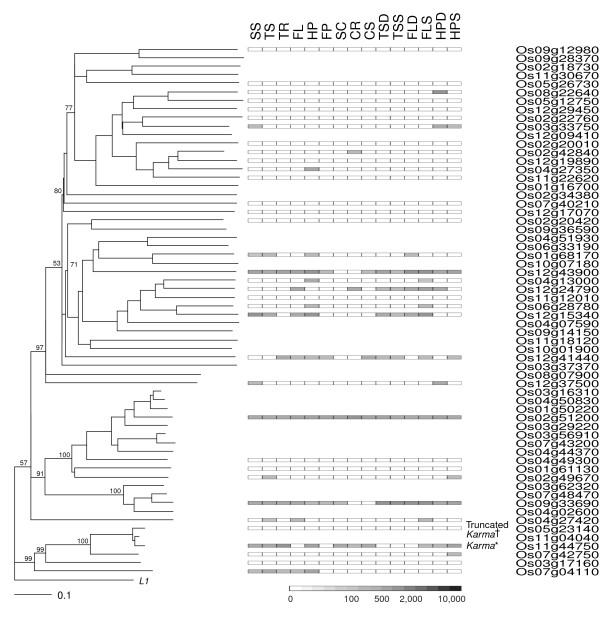
Degrees of lineage-specific transcription in the LINE superfamily. The phylogenetic tree was generated from a multiple alignment of conceptually translated sequences by using neighbor-joining methods and rooted with human *L1*. Bootstrap values were calculated from 1,000 replicates. Sample numbers are identical to those in Table 2. Shades of gray indicate the magnitude of transcription signals, which are based on microarray hybridization signals without units. Names of previously reported members are shown. *Previously reported members with transcription or transposition. ^† ^Previously reported inactivate members. LINE, long interspersed element.

The type I TE-related genes can be classified into two groups according to the presence or absence of long terminal repeats (LTRs). TE-related genes without LTRs belong to the long interspersed elements (LINEs) type, which may encode retrotransposase and mobilize noncoding short interspersed elements (SINEs). Only 34 LINE-type TE-related genes were identified in rice (Table 1). We found a relatively small portion (usually below 20%) of this family transcribed (Figure 3). One rice LINE-type retrotransposon named *Karma *with active transposition has been reported [20]; its transcriptional activity was detected in a wide range of organs and cultured cells. A 5'-truncated version of *Karma *was also identified in the rice genome [20], which lacks transcriptional activity in all samples we tested (Figure 3).

LTR-type TE-related genes belong to two superfamilies, namely Ty1/*copia *and Ty3/*gypsy*, which are both ubiquitous throughout plants and believed to have contributed significantly to the evolution of genome structure and function [10]. Both families are quite diverse in rice, with Ty3/*gypsy *elements outnumbering Ty1/*copia *elements [48]. Our expression data indicate that both families are similarly transcribed at low levels at around 25% in most samples, but there are members in both families with strong transcription in widespread tissues. However, they are spread in different clades with only remote similarity (Additional data files 1 and 2). A few active LTR retrotransposons have been reported in rice. Among them, *Tos17 *is the best characterized and is known to exhibit active transposition in tissue culture [19]. We found active transcription of *Tos17 *not only in cultured cells but also in a wide range of organs (Additional data file 1), suggesting that tissue culture may provide a way to propagate somatic transposition events to progeny. Sireviruses are a plant-specific lineage of the Ty1/*copia *retrotransposons that interact specifically with proteins related to dynein light chain 8 [50]. We found one member of this lineage with ubiquitous strong transcription and several others with transcription in selected rice samples (Additional data file 1).

A large number of type I TE-related genes have not yet been further classified (Table 1). We detected transcription of a smaller proportion of this group of genes than for Ty1/*copia *and Ty3/*gypsy *superfamilies.

### Transcription of type II TE-related genes

Type II TE-related genes are in general more actively transcribed than type I TE-related genes. Different from type I, type II TE-related genes are highly variable among major superfamilies with respect to transcriptional activity. Whereas CACTA and MULE superfamilies are actively transcribed, *hAT*-like, *PIF*/*Pong*-like, *Mariner*-like, and *Helitron*-like superfamilies have transcriptional activities similar to or lower than those of type I TE-related genes.

**Figure 4:**
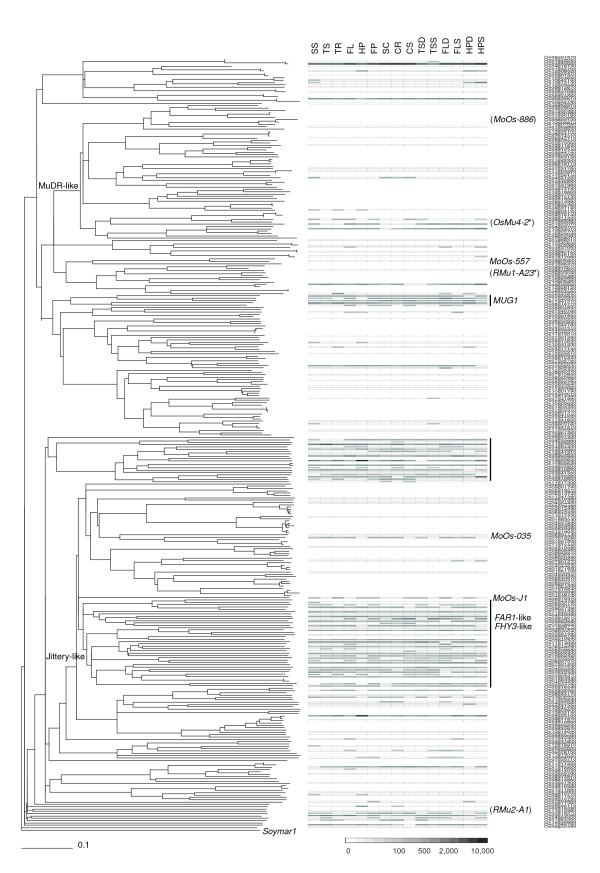
Degrees of lineage-specific transcription in MULE superfamily (excluding the *TRAP*-like class). The phylogenetic tree was generated from a multiple alignment of conceptually translated sequences by using neighbor-joining methods and rooted with soybean *Soymar1*. Bootstrap values were calculated from 1,000 replicates. Sample numbers are identical to those in Table 2. Shades of gray indicate the magnitude of transcription signals, which are based on microarray hybridization signals without units. Names of previously reported members are shown. Names in parenthesis indicate members not covered by microarray. Transcriptional active clades are highlighted by bars. *Previously reported members with transcription or transposition.

*Mutator*-like superfamily (MULE) is one of the first groups of identified transposases with a few reported transcriptionally active members in rice [27]. There are 607 autonomous members of this superfamily (Table 1), which has one of the strongest transcription levels, at 35% to 40% in each sample (Figure 4). The MULEs can be further divided into three branches: *MuDR*-like, *Jittery*-like, and *TRAP*-like [27]. The *TRAP*-like branch may have recently been amplified, and high similarity among family members has resulted in lack of unique oligo probes with which to examine their expression profiles. Interestingly, we have found at least three clades with clear active transcription in *MuDR*-like and *Jittery*-like branches (Figure 4). The one highly transcribed clade in the *MuDR*-like branch included *MUG1*, an evolutionarily conserved MULE sequence found in diverse angiosperms and a candidate for categorization as a domesticated transposase-related gene [36]. The larger, highly transcribed clade in the *Jittery*-like branch includes homologs to *Arabidopsis *genes *FAR1 *and *FHY3*, both of which are transposon-derived genes with demonstrated host function as transcription factors downstream of phytochrome A [32,51,52]. There are no reports on any members of the other highly transcribed clade in the *Jittery*-like branch, which has rampant transcription (Figure 4, middle).

The CACTA superfamily is a diverse group of high-copy repetitive genes in grasses [53,54]. CACTA transposons with active transcription or even transposition have been reported in rice and other grass genomes [54-57]. A total of 2,276 intact CACTA transposase-coding genes are identified in rice, making it the largest superfamily in type II TE-related genes (Table 1). The CACTA superfamily is also highly active, with more than 40% transcribed in each sample. Several clades with active transcription were identified (Additional data file 4). Among them, two clades include over 20 members. No members within these actively transcribed CACTA transposons have previously been characterized.

**Figure 5:**
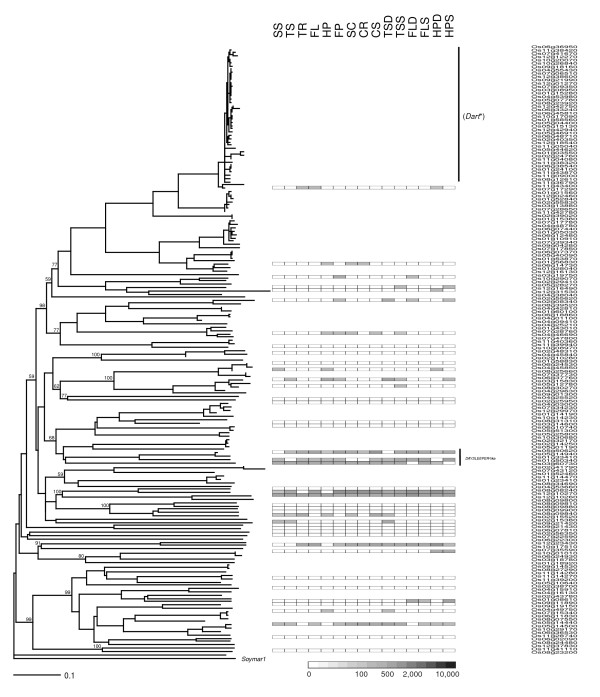
Degrees of lineage-specific transcription in *hAT*-like superfamily. The phylogenetic tree was generated from a multiple alignment of conceptually translated sequences by using neighbor-joining methods and rooted with soybean *Soymar1*. Bootstrap values were calculated from 1,000 replicates. Sample numbers are identical to those given in Table 2. Shades of gray indicate the magnitude of transcription signals, which are based on microarray hybridization signals without units. Names of previously reported members are shown. *Previously reported members with transcription or transposition.

The *hAT*-like superfamily is another widespread superfamily in grasses [58]. It is a medium-sized superfamily in rice with 184 autonomous members (Table 1). About 20% of this superfamily is transcribed in a single sample (Figure 5). Interestingly, we found a small clade of four genes that exhibited relatively uniform and strong transcription across a wide range of samples. A sequence comparison indicates that these genes have high similarity with a recently identified domesticated *Arabidopsis *transposase *DAYSLEEPER*, which is a pleiotropic regulator of development through its specific DNA-binding activity [33]. There is one reported *hAT*-like transposon group in rice, *Dart*, which is capable of active transposition in plants [24,59]. Sequence analysis indicates that *Dart *is a recently amplified clade with 30 almost identical members. Although no oligonucleotide probes have been developed to represent individual members, there are a few probes that can detect all or most of them. Clear hybridization signals have been found for these probes in all shoot and cell culture samples. This finding suggests that some or all members of *Dart *are highly transcribed in a large number of rice samples.

**Figure 6:**
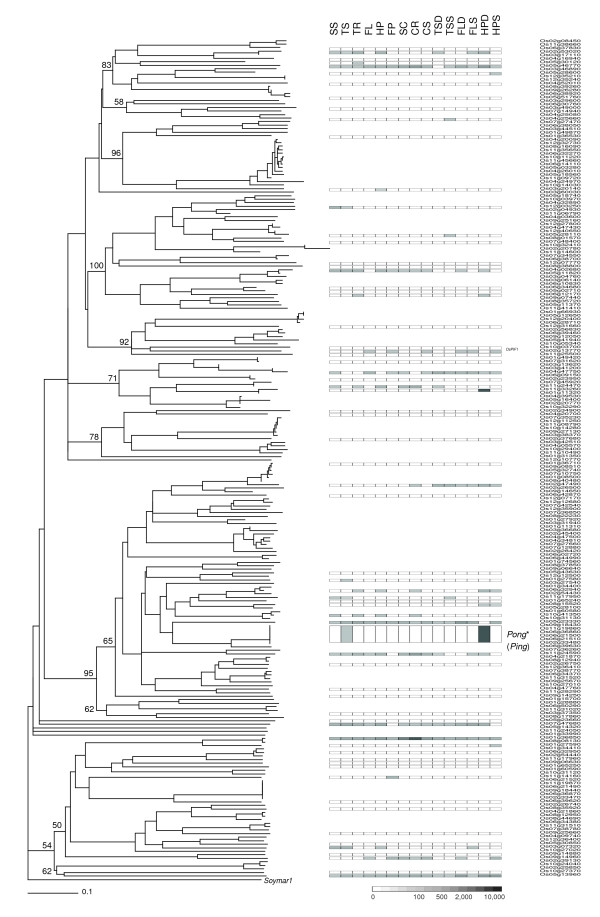
Degrees of lineage-specific transcription in *PIF*/*Pong*-like superfamily. The phylogenetic tree was generated from a multiple alignment of conceptually translated sequences by using neighbor-joining methods and rooted with soybean *Soymar1*. Bootstrap values were calculated from 1,000 replicates. Sample numbers are identical to those in Table 2. Shades of gray indicate the magnitude of transcription signals, which are based on microarray hybridization signals without units. Names of previously reported members are shown. Names in parenthesis indicate members not covered by the microarray. *Previously reported members with transcriptional or transpositional activity.

Both *PIF*/*Pong*-like and *Mariner*-like TE-related genes are autonomous partners of nonautonomous miniature inverted repeat transposable elements (MITEs), which are ubiquitous in the rice genome [12]. Low proportions of both families have detectable transcription (<20%) in each sample (Figure 6 and Additional data file 4). Two transpositionally active *PIF*/*Pong*-like elements were recently reported: maize *PIF *and rice *Pong *[22,23,60]. Interestingly, the rice homolog of *PIF*, namely *OsPIF1 *[60], was not expressed in any samples (Figure 6). There are six almost identical *Pong *elements in the rice genome, which are represented by a single probe in the microarray. This probe detected transcription activity in tillering shoot and drought-exposed panicles only (Figure 6), suggesting rigorous regulation at the transcriptional level for members of this family. We did not detect any transcriptional activity of the *Pong *element in cultured cells. The *Mariner*-like superfamily has a much smaller member size [61]; this superfamily includes a small proportion of transcribed genes, similar to that for the *PIF*/*Pong*-like superfamily (Additional data file 4).

A recently identified unique type II TE superfamily, *Helitron*-like, is relatively under-characterized in the rice genome [62]. Strikingly, *Helitron*-like transposons have the potential to move and shuffle genes or exons in maize [13,14]. In rice, we found only one member with transcriptional activity in all the samples. There is no other *Helitron*-like transposon among the seven examined ones with transcriptional activity in any samples (Additional data file 5).

We were unable to further classify another 787 type II TE-related genes into any superfamilies (Table 1). Interestingly, a large percentage (>40% out of 128 with unique oligomer probes) was found to be transcribed.

### Transcription of Pack-MULE

**Figure 7:**
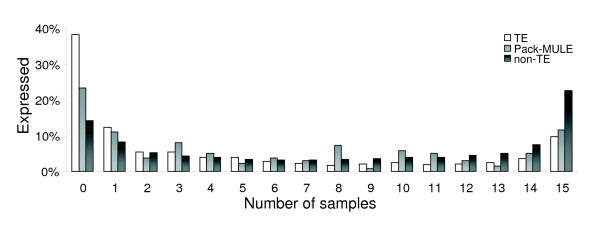
Summary of expression of Pack-MULEs in comparison with other TE-related and non-TE-related gene models. Levels of transcription can be inferred based on how often expression was detected in the different samples for each group. TE, transposable element.

Genes or exons can be transduplicated by MULEs [9,63], which have recently been suggested to be important facilitators of the evolution of genes in higher plants, and have therefore been termed Pack-MULE [37]. However, a detailed sequence analysis suggests that the products of this process are more likely to be pseudogenes than novel functional genes [39]. To gain better insight into this group, we examined their transcriptional activities using microarray analysis, because transcription is usually a prerequisite for biologic function of a protein-coding gene. By testing the transcription of recently identified 137 Pack-MULEs on chromosomes 1 and 10 that are covered by our microarray [37], we found that the transcription rates of Pack-MULEs fall between those of TE-related gene models and non-TE-related gene models (Figure 7), being slightly closer to those of TE-related gene models. On the other hand, more Pack-MULEs are transcribed in several samples than for TE-related gene models and non-TE-related gene models (Figure 7).

### Association of transcription with DNA and histone modification

TEs, including TE-related ORF encoding genes, are under multiple levels of epigenetic control, including DNA methylation and histone modifications [26]. In Arabidopsis, DNA methylation and histone H3 lysine-9 methylation (H3K9m) correlates with the silencing of TEs, and histone H3 lysine-4 methylation (H3K4m) is associated with transcribed genes [64]. However, H3K4m is also found in silenced genes and therefore may not always be a marker for active transcription [65].

**Figure 8:**
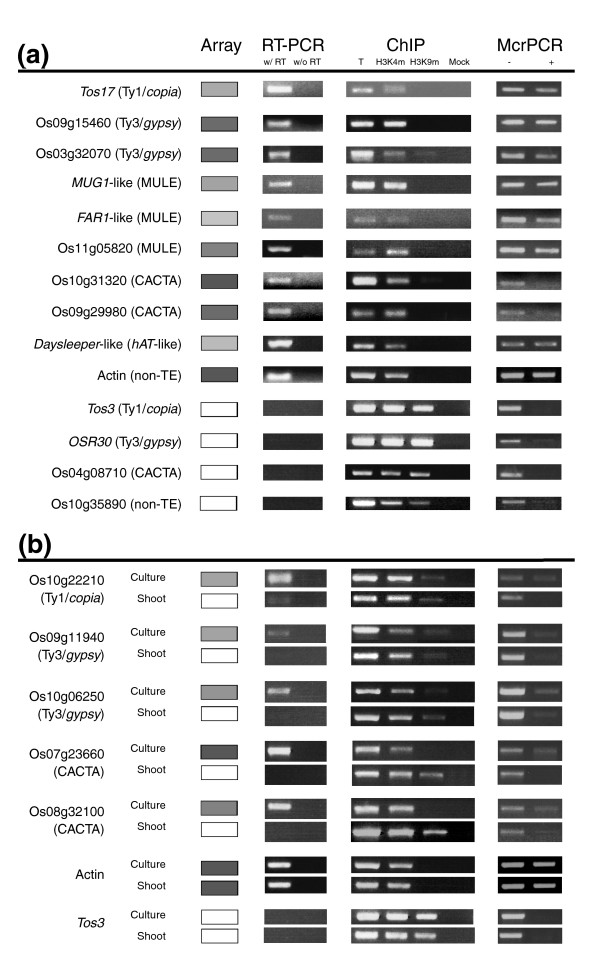
Chromatin-level modifications of TE-related genes. Reverse-transcribed cDNA, DNA from ChIP, and McrBC-digested genomic DNA were amplified by PCR for TE-related genes **(a) **with and without transcription in seedling shoots, and **(b) **with transcription in cultured cells but not seedling shoots. Primers corresponded to transcribed ORFs. Mock RT-PCR was performed without reverse transcriptase (w/o RT). ChIP was carried out with histone H3 anti-dimethyl lysine-4 (H3K4m) or anti-dimethyl lysine-9 (H3K9m) antibodies together with total DNA input (T) and no antibody (Mock) controls. McrPCR was performed on McrBC digested (+) and untreated (-) total genomic DNA. Actin was used as a positive control and Os10g35890, a gene of unknown function without transcription in seedlings, as a negative control. The same gray scale was used to indicate magnitude of transcription signals from microarray (Array). ChIP, chromatin immunoprecipitation; ORF, open reading frame; PCR, polymerase chain reaction; TE, transposable element.

To determine whether transcribed TE-related genes have different chromatin modification status, we selected nine transcribed and three silenced TE-related genes, including both autonomous TE genes and TE-derived genes, in order to assess histone and DNA methylation (Figure 8a). These are *Tos17* and *Tos3* of the Ty1/*copia* superfamily; Ty3/*gypsy* elements Os09g15460, Os03g32070 and *OSR30*; MULE superfamily DNA transposons *MUG1*, *FAR1*-like and Os11g05820; CACTA DNA transposons Os10g31320, Os09g29980 and Os04g08710; and *DAYSLEEPER*-like from the *hAT*-like superfamily. Seedling shoot samples were used for all analyses discussed here. To verify transcription independently, we used PCR to amplify reverse-transcribed cDNA (RT-PCR). Transcript accumulation assayed by RT-PCR is in general consistent with microarray results (Figure 8a). Using chromatin immunoprecipitation (ChIP) analysis, we found that only silenced genes were associated with high levels of H3K9m. H3K4m was significant for all genes examined, regardless of whether they were transcribed or silenced (Figure 8a). Similar to H3K9m, only silenced genes were heavily methylated at the DNA level (at cytosine, by McrBC digestion assay; Figure 8a). These data imply that levels of H3K9m and DNA methylation were lower in transcribed TE-related genes. Similar correlations of histone and DNA methylation with transcription were also found in non-TE-related genes (controls in Figure 8a). Furthermore, no distinction was found between autonomous TE genes and TE-derived genes from these data.

To explore these relationships further, we selected five TE-related genes with transcription in cultured cells but not in seedling shoots: the Ty1/*copia* retroelement Os10g22210; Ty3/*gypsy* retrotransposons Os09g11940 and Os10g06250; and CACTA DNA transposons Os07g23660 and Os08g32100 (Figure 8b). Three of these five genes were associated with higher levels of H3K9m in shoots (silenced) as compared with in cultured cells (transcribed), according to ChIP-PCR analysis. Levels of H3K4m did not exhibit a clear difference between shoots and cultured cells (Figure 8b). DNA methylation was reduced in three genes in cultured cells compared with shoots (Figure 8b). Thus, lower levels of DNA methylation and H3K4m tend to accompany TE-related gene transcription under developmental regulation.

It has been shown that small RNAs derived from repetitive genome sequences repress transcription by means of RNA interference in *Arabidopsis *[16]. Small RNAs, both microRNAs (miRNAs) and small interfering RNAs (siRNAs), have also been identified in rice, albeit at a small scale [66,67]. Sixteen out of a total of 44 predicted siRNAs have at least one TE-related gene as their target gene [66], whereas few miRNA have a TE-related gene target [67]. For the five target TE-related genes covered by microarray, we found active transcription for only one. It is interesting to note that for siRNAs targeting multiple genes, the transcriptional profiles of these target genes may not be at all similar. For example, siRNA P96-E12 has two targets: Os07g10770 (a cellulose synthase) and Os01g05370 (a Ty1/*copia *family retrotransposon). The cellulose synthase gene has strong transcription in almost all samples we profiled. In contrast, the retrotransposon target does not exhibit transcription in any sample.

### Upstream gene transcription affects TE-related gene transcription

**Figure 9:**
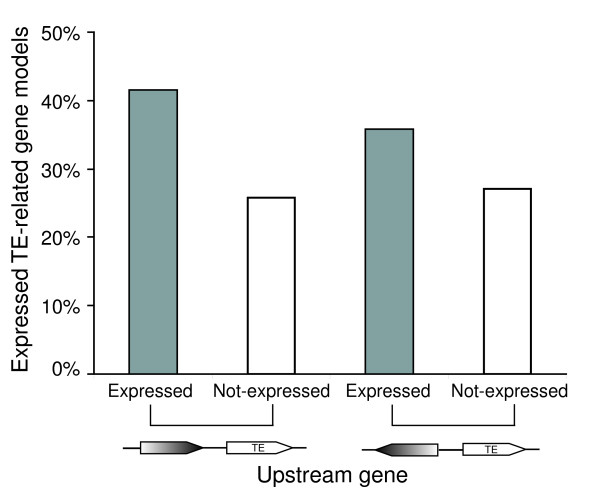
Effects of relative orientation of upstream genes on transcription of downstream TE-related genes. All TE-related genes were divided into two groups according to the relative orientations of themselves and upstream genes. Portions of transcribed TE-related genes were calculated for those with transcribed upstream genes and those with silent upstream genes in both groups. TE, transposable element.

It was recently reported in *Arabidopsis*, as well as in several other eukaryotes, that some adjacent genes tend to have co-expression patterns [68-71]. Readthrough of TEs derived from upstream genes is also reported in isolated studies [41,72,73]. We therefore suspected that transcription of neighboring genes might influence the transcription of a TE-related gene. To test this hypothesis, we calculated the frequency of transcribed TE-related genes relative to the transcriptional activity of neighboring genes. Two scenarios were considered: the upstream gene and the downstream TE-related gene were in the same orientation (or the same strand); and these two were in opposite orientations. In both cases, there was a clear positive association between gene transcription and the neighboring TE-related gene transcription (Figure 9). However, the effect was more significant if the non-TE-related and TE-related genes were in the same orientation. An increase of 16% of downstream transcription was found when transcribed upstream genes were in the same orientation (*P *< 10^-16^, by Welch two-sample *t*-test). In the case of opposite orientation, an increase of 9% in transcription level was found (*P *< 10^-16^). By comparing the effects of transcribed upstream gene orientation in these two scenarios, we found that the same orientation corresponded to 6% more expression than the other scenario (*P *< 10^-7^). There is no clear distinction between the two scenarios for TE-related genes with untranscribed upstream genes (26% versus 27%; *P *= 0.3). The orientation of downstream non-TE-related genes did not significantly affect the transcription of upstream TE-related genes.

### Functions of *cis*-elements in transcription

**Figure 10:**
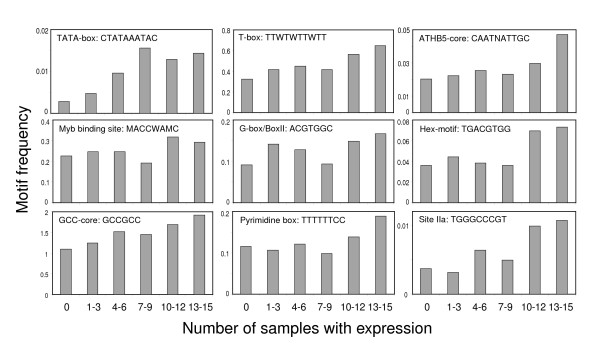
Motifs with enrichment in transcribed TE-related gene promoters. Genes were grouped according to the number of samples that show transcriptional activity. Enrichment was measured as the frequency of a motif in gene promoters of a certain group. TE, transposable element.

To explore further the possible underlying mechanisms that control the transcription of TE-related genes, we attempted to identify possible involvement of *cis* elements in transcription. To this end, we searched for enrichment of *cis* elements in the promoter regions of transcribed TE-related genes. We grouped TE-related genes based on the number of samples with transcription and searched for frequency of occurrence of all reported *cis* elements within each group. Among 439 reported elements in plants [74], nine of them exhibited marked enrichment in TE-related genes with active transcription (Figure 10), whereas no element was found with similar enrichment patterns from randomized datasets. In addition, most of these elements were found by searching for enrichment in active members in Ty1/*copia*, Ty3/*gypsy*, or the CACTA superfamily. TATA box was identified, which is usually found in the 5'-upstream region of eukaryotic genes and is critical for accurate initiation of transcription [75]. The T-box is part of the scaffold/matrix attachment region, which was recently found to regulate the transcription of nearby genes in *Arabidopsis *[76]. We also identified the enrichment of motifs (G-box, Myb binding site, and ATHB5-core) for the major plant transcription factor families (bHLH, Myb, and homeodomain-leucine zipper). In addition, enrichment was also detectable from the light response motifs Hex-motif, pathogen response motif GCC-core, gibberellin response motif Pyrimidine-box, and meristem specific motif site IIa.

## Discussion

### Transcription profiles of TE-related genes in rice

TEs account for an overwhelming proportion of plant genomes. To ensure the viability of their host and hence their own survival, the transposition of TEs should be tightly controlled [17]. Transcribed autonomous TEs among TE-related genes have the potential to self-activate or activate transcription of related nonautonomous TEs. Transcriptional regulation is therefore one major control step used by plants, but it remains insufficiently understood. The recently available rice genome sequence has enabled us to characterize TE-related gene transcription on a genome-wide scale.

Using 70-mer oligonucleotide microarrays covering more than 2,000 rice TE-related genes, we surveyed the transcription profiles under a wide range of organ samples under various conditions. Considering that TE-derived cellular genes are relatively rare, autonomous TEs probably contribute to most of these TE-related genes. Genome profiling revealed that 25% to 30% of the TE-related genes were transcribed in one sample, which was much lower than the corresponding percentage of non-TE-related genes (Figures 1 and 2). Moreover, TE-related genes differed from their non-TE-related counterparts in two additional aspects. First, TE-related genes tended to be transcribed in only a subset of organs or developmental stages, whereas non-TE-related genes had transcription in more samples on average (Figure 1 and Figure 2). Second, transcribed TE-related genes exhibited weaker transcription overall compared with non-TE-related genes in all of the samples we profiled (Figure 2). It worth noting that our estimation of TE-related gene transcription was biased toward low-copy elements, because it was difficult to distinguish transcripts among recently duplicated high-copy TE-related genes, which share high sequence similarity within clades. It has been reported in *Arabidopsis *and *Drosophila *that the activity of TE elements may reduce as the copy number increases [77,78]. Therefore, we expect the transcriptional activity of those high-copy TE-related genes will be lower than for low-copy ones.

Among TE-related genes, a smaller proportion of type I than type II genes were transcribed (Figure 1a), a discrepancy that resulted primarily from the strong transcription of MULE and CACTA superfamilies as well as unclassified type II members. It is interesting to note that all TE-related gene superfamilies with potential to severely expand, including all type I TE-related genes and *PIF*/*Pong*-like, *Mariner*-like and *Helitron*-like type II TE-related genes, were more tightly controlled at the transcription level. Type I TE-related genes are amplified through a copy-and-paste mechanism [79]. *PIF*/*Pong*-like and *Mariner*-like superfamilies regulate the activity of MITEs, which dominate the rice genome [12]. Members of the *Helitron*-like superfamily go through a unique rolling cycle replication to rapidly amplify themselves [62].

Many TE-related genes exhibit organ-specific, growth stage-specific, and stress-specific expression profiles in our collection of samples. These genes exist in all superfamilies, as shown in Figures 3 to 7. A number of them, again from various superfamilies of both type I and type II TE-related genes, exhibit clear induction in cultured cells, in certain organs, or in certain stress challenged organs (Figure 2). The precise biologic significance for this observation remains to be elucidated.

It is important to note that transcriptional activity does not necessarily correspond to transpositional activity. Transcription is just the first of several steps required for the transposition of type I and type II TEs [79,80]. Active transcription and even translation of TE-related genes has been reported in several isolated cases [28], but only in a few cases was transposition actually confirmed by observed copy number change [20]. A two-step regulatory mechanism was therefore proposed for retrotransposons [20]. In this model, some elements may have slipped the leash of transcriptional gene silencing [25]. Nevertheless, they can be controlled by post-transcriptional gene silencing [18]. We observed transcription of all major TE-related gene superfamilies in rice, but it is probable that most of them, if not all, are not actively transpositioned. It is therefore likely that such a two-step regulation exists not only for retrotransposons but also for other classes. Post-transcriptional regulation, which is still largely unexplored, is thought to repress transposition activity further [81].

### Transcription of domesticated TE-related genes in the rice genome

It is well accepted that some TE-related genes have actually acquired host functions and play physiologic roles in the host. They can either be derived from TEs or include hijacked TEs or TE fragments by cellular genes. Not surprisingly, we have discovered active transcription of all potential domesticated TE genes previously described in *Arabidopsis *and rice. Interestingly, domesticated TE genes tend to be within actively transcribed TE gene clades. The rice homologs of the two reported cases of domesticated transposons in *Arabidopsis*, namely *FAR1*/*FHY3 *and *DAYSLEEPER*, were located in two actively transcribed clades. *MUG1*, a putative domesticated gene revealed by cross-species sequence comparison analysis, was shown to be transcribed from our data and located within an actively transcribed clade. These examples may suggest that actively transcribed clades of TE-related genes are a rich source for domesticated TE genes. In fact, several other actively transcribed clades have been observed, especially for the MULE and CACTA superfamilies, from our analysis (Figures 4 and 5). It is reasonable to suspect that those transcriptionally active clades may contain genes co-opted by hosts to serve adaptive functions. This notion will be worth testing in future research. Clearly, the combination of transcriptional analysis with phylogenetic analysis is instrumental in identifying those TE-derived genes with adapted host function.

A specific mechanism for the evolution of new genes by mobile DNA elements is through their ability to acquire and fuse fragments of genes to new genomic locations, as represented by Pack-MULE [37]. By exploring the transcriptional activity of a subset of Pack-MULEs, we have shown that their transcriptional activity falls in between the levels of TE-related and non-TE-related gene models (Figure 7). This result suggests that many of them might not have biologic functions, and both pseudogenes and evolving new functional genes exist among these annotated Pack-MULEs. Alternatively, functional diversification of recently evolved genes may be another explanation, because newly formed genes usually have more specific expression profiles [82].

### Mechanisms controlling TE-related genes transcription

The presence of such a diverse array of transcribed TE-related genes raises questions regarding the mechanisms that control the transcription. At the chromatin level, we found that actively transcribed TE-related genes have reduced levels of H3K9m and DNA methylation. This finding indicates that proper chromatin modification status is usually required for transcription of TE-related genes. However, histone and DNA modifications are unlikely to be efficient markers for distinguishing between autonomous TE genes and TE-derived cellular genes.

Consistent with the existence of chromatin-level control, we found that transcribed TE-related genes tend to be located near to transcribed neighboring genes. It is possible that the status of a chromatin domain is marked by histone and DNA modifications. Such chromatin status affects a few genes located in the same or neighboring chromatin domains. The orientation of upstream genes affects downstream TE-related gene transcription (Figure 9). If both genes are in the same orientation, then the downstream TE-related gene would have a greater chance of being transcribed. Readthrough of TE-related genes derived from upstream genes may account for this difference, besides possible chromatin effects.

Small RNA has been suggested to be a key regulator to silence TE elements transcriptionally and post-transcriptionally [18,81]. However, only a few examples were found in our dataset. Small RNAs are known to be highly abundant in the *Arabidopsis *genome [83], whereas their counterparts in rice are yet to be discovered. A full catalog of small RNAs in rice will provide a better picture of their role in TE transcription.

Another possible mechanism controlling TE transcription is the existence of *cis *elements in their promoter regions. Examples have been found previously for LTR retrotransposons, which employ alternating *cis *elements present in their LTRs [29,84-86]. Here, we identified nine *cis *elements that were clearly enriched in the promoter regions of transcribed TE-related genes. Among them, both basic transcription-related *cis *elements and elements that respond to developmental or environmental regulation are found to be enriched in the upstream regions of those transcribed TE-related genes (Figure 10). In addition, these enriched *cis *elements are probably not limited to a certain superfamily but rather widely spread in several superfamilies. Taken together, our data show that transcription of TE-related genes, mostly autonomous TE genes, in rice is a complex process, which is controlled, at least in part, by chromatin-level regulation and *cis *elements in promoters.

## Materials and methods

### Microarray analysis

The rice 70-mer oligonucleotide set was described previously [44]. Briefly, 70-mer oligonucleotides were designed based on a combination of FGENESH predicted genes from an improved shotgun sequence [2] and the available full-length cDNAs and ESTs [87]. Designed 70-mer oligonucleotides correspond to the sequence within the coding region of genes, and the design was corrected for such factors as oligo cross-hybridization, uniform TM value, GC content, and hairpin/stem nucleotide number. All oligonucleotides were remapped to TIGR rice genome annotation version 3.1 genes [3] using BLAST. We requested greater than 90% alignment of a 70-mer oligonucleotide probe to a gene during the remapping. Moreover, only those 70-mer probes without a greater than 80% second-best aligned gene were considered to be free from cross-hybridization. These criteria were selected because a mismatch of 20% removes more than 90% of the hybridization signals, whereas a 10% mismatch retains at least half of the hybridization signals [88].

TE-related genes were identified in accordance with TIGR annotation, with supplemental literature review of published TE-related genes. A total of 2,191 TE-related genes are represented by at least one oligonucleotide free from cross-hybridization. In addition, there are 1,966 70-mer oligonucleotides mapped to several but only TE-related genes. These oligonucleotides represent another 9,396 TE-related genes.

Oligonucleotides were custom synthesized by Operon Biotechnologies Inc. (Huntsville, AL, USA) and printed onto poly-L-lysin coated microscope slides using a contact microarrayer. The same recommended set of 12 unique negative control 70-mer oligonucleotides based on heterologous genes [89] were included in all slides. There were 240 negative control spots on each slide.

### Microarray data and plant materials

Microarray experiments and detailed rice sample preparation were described previously [44-47]. Samples include organs harvested under normal growth conditions (seeding stage shoot, tillering stage shoot, tillering stage root, heading stage flag leaf, heading stage panicle, and filling stage panicle), organs under conditions of salinity or drought (tillering stage shoot, heading stage flag leaf, and heading stage panicle), and cultured cells (suspension-cultured cells, somatic root in culture, and somatic shoot in culture). A summary is provided in Table 2. The microarray data discussed in this publication have been deposited in NCBI Gene Expression Omnibus [90] and are accessible through GEO series numbers GSE2360, GSE2691, GSE6533, and GSE6552.

### Microarray data processing

Microarray spot intensity signals were acquired using Axon GenePix Pro 3.0 software package (Molecular Devices, Sunnyvale, CA, USA). To identify and remove systematic sources of variation, including dye and spatial effects, spot intensities from the GenePix Pro output files of all repeats of a given sample pair were normalized using limma, a software package for the analysis of gene expression microarray [91]. This normalization process identified and ameliorated spatial, intensity-based, and dye-specific artifacts using multiple step corrections. To determine objectively whether a gene exhibited significant expression in a given sample, we followed a method that relied on negative control spots and data reproducibility [44]. To estimate nonspecific hybridization, a distribution of normalized intensities was obtained from the subset of negative control spots present on each array slide. From this distribution, we chose an intensity cutoff at which less than 10% of the distribution was greater than or equal to this threshold. Expression of a gene was only considered detectable if it was above the threshold in two or more repeats out of the three. These criteria had been demonstrated suitable for oligonucleotide arrays with an error rate range of 1% to 3% false negatives [44]. RT-PCR results and independent analysis using different microarrays and statistical approaches [43] further supported this threshold.

### Sequence analysis

TE family classification was according to TIGR annotation [3]. Hand analysis led to the identification of another 208 TE-related genes according to published sequences and BLAST search. Multiple sequence alignments were conducted using Clustal W [92]. The weighing matrix used was Gonnet Pam 250 with the penalty of gap opening 10 and gap extension 0.2. Phylogenetic trees were generated based on the neighbor-joining method, using PAUP* version 4.0b10 with default parameters [93].

### Cluster analysis

Cluster analysis was applied to all TE-related genes and 1,353 randomly selected non-TE-related genes showing expression in at least one sample. Average normalized log-transformed expression intensities were subjected to cluster analysis. For hierarchical clustering, Pearson correlation was used to compute similarities, and a complete linkage clustering algorithm was used. Cluster analysis was performed using the software Cluster [94] and visualized using custom scripts.

### RT-PCR analysis

Total RNA was extracted from independently prepared rice seedling shoots using Qiagen RNeasy kit (Qiagen, Valencia, CA, USA). After DNase I treatment, total RNA was used for cDNA synthesis using Superscript II (Invitrogen, Carlsbad, CA, USA) in accordance with the manufacturer's protocol. PCR primers were designed according to sequence using Primer3 [95]. The amplification reaction was carried out for 35 cycles and at an annealing temperature of 55°C. Products were separated by 1% agarose gel electrophoresis. Negative controls using mock cDNA synthesis products without reverse transcriptase were included for all genes to detect potential genomic DNA contamination.

### Histone and DNA methylation

ChIP was carried out as described elsewhere [64] using seedling shoots and cultured cells. Histone H3 anti-dimethyl lysine-4 or anti-dimethyl lysine-9 antibodies (Upstate, Avon, NY, USA) were used to precipitate genomic DNA, which was resuspended in water for PCR analysis. The same PCR and gel electrophoresis conditions were used as for RT-PCR analysis.

Methylation of DNA was assessed by McrBC digestion following a previously published protocol [81]. Genomic DNA was isolated from seedling shoots and cultured cells using Qiagen DNeasy plant kit and divided into two equal samples. One sample was digested with McrBC, a methylation-dependent restriction enzyme that cuts the sequence A/G 5 mC (New England Biolabs, Beverly, MA, USA). Both digested and untreated samples were subject to PCR amplification as described previously. Successful amplification after digestion indicates lack of methylation.

### Motif search

The genome sequences 2 kilobases upstream of the annotated translation start site were retrieved from the TIGR database. Both DNA strands were searched for known plant motifs using the PLACE database [74]. Enrichment levels were further calculated using custom scripts [45].

## Additional data files

The following additional data are available with the online version of this paper. Additional data file 1 shows degrees of lineage-specific transcription in the Ty1/*copia *superfamily. Additional data file 2 shows degrees of lineage-specific transcription in the Ty3/*gypsy *superfamily. Additional data file 3 shows degrees of lineage-specific transcription in the CACTA superfamily. Additional data file 4 shows degrees of lineage-specific transcription in the *Mariner *superfamily. Additional data file 5 shows degrees of lineage-specific transcription in the *Helitron *superfamily.

## Supplementary Material

Additional data file 1 The phylogenetic tree was generated from a multiple alignment of conceptually translated sequences by using the neighbor-joining methods and rooted with human *L1*. Bootstrap values were calculated from 300 replicates. The sample numbers are identical to those in Table 2. Shades of gray indicate the magnitude of transcription signals, which are based on microarray hybridization signals without units. Names of previously reported members are shown. Names in parenthesis indicate members not covered by microarray. *Previously reported members with transcription or transposition. Click here for file

Additional data file 2 The phylogenetic tree was generated from a multiple alignment of conceptually translated sequences by using neighbor-joining methods and rooted with human *L1*. Bootstrap values were calculated from 300 replicates. Sample numbers are identical to those in Table 2. Shades of gray indicate the magnitude of transcription signals, which are based on microarray hybridization signals without units. Names of previously reported members are shown. Names in parenthesis indicate members not covered by microarray. *Previously reported members with transcription or transposition. Click here for file

Additional data file 3 The phylogenetic tree was generated from a multiple alignment of conceptually translated sequences by using neighbor-joining methods and rooted with soybean *Soymar1*. Bootstrap values were calculated from 300 replicates. Sample numbers are identical to those in Table 2. Shades of gray indicate the magnitude of transcription signals, which are based on microarray hybridization signals without units. Names of previously reported members are shown. Names in parenthesis indicate members not covered by microarray. *Previously reported members with transcription or transposition. Click here for file

Additional data file 4 The phylogenetic tree was generated from a multiple alignment of conceptually translated sequences by using neighbor-joining methods and rooted with soybean *Soymar1*. Bootstrap values were calculated from 300 replicates. Sample numbers are identical to those in Table 2. Shades of gray indicate the magnitude of transcription signals, which are based on microarray hybridization signals without units. Click here for file

Additional data file 5 The phylogenetic tree was generated from a multiple alignment of conceptually translated sequences by using neighbor-joining methods and rooted with *C. elegance CeHEL1*. Bootstrap values were calculated from 300 replicates. Sample numbers are identical to those in Table 2. Shades of gray indicate the magnitude of transcription signals, which are based on microarray hybridization signals without units. Click here for file

## Figures and Tables

**Table 1:** Summary of annotated TE-related genes in rice and coverage by (cross-hybridization free) microarray probes

	Number of TEs in TIGR	Number of TEs in TIGR and literature review	Covered by microarray
Type I			
Ty1/*copia*	1,273	1,469	235
Ty3/*gypsy*	3,904	4,218	362
LINE	56	62	34
Undetermined	4,158	3,744	691
Subtotal	9,391	9,493	1,322
Type II			
*hAT*-like	13	184	42
CACTA	2,392	2,276	231
*MULE*	452	607	155
*PIF*/*Pong*-like	122	238	67
*Mariner*-like	48	48	15
*Helitron-like*	0	19	7
undetermined	999	787	128
Subtotal	4,026	4,159	645
Unclassified	779	752	224
Total^a^	14,196	14,404	2,191

^a^The two subtotals plus Unclassified. TE, transposable element.

**Table 2:** Summary of rice samples used in this study

Sample	Abbreviation
Seedling shoot	SS
Tillering stage shoot	TS
Tillering stage root	TR
Flag leaf	FL
Heading panicle	HP
Filling panicle	FP
Suspension cultured cells	SC
Somatic root in culture	CR
Somatic shoot in culture	CS
Tillering stage shoot under drought stress	TSD
Tillering stage shoot under salt stress	TSS
Flag leaf under drought stress	FLD
Flag leaf under salt stress	FLS
Heading panicle under drought stress	HPD
Heading panicle under salt stress	HPS
