# Supplementary figures and images for: A genome-wide transcriptional activity survey of rice transposable element-related genes
Source: Genome Biol. 2007 Feb 27;8(2):R28. doi: 10.1186/gb-2007-8-2-r28 (PMC1852403; doi:10.1186/gb-2007-8-2-r28)

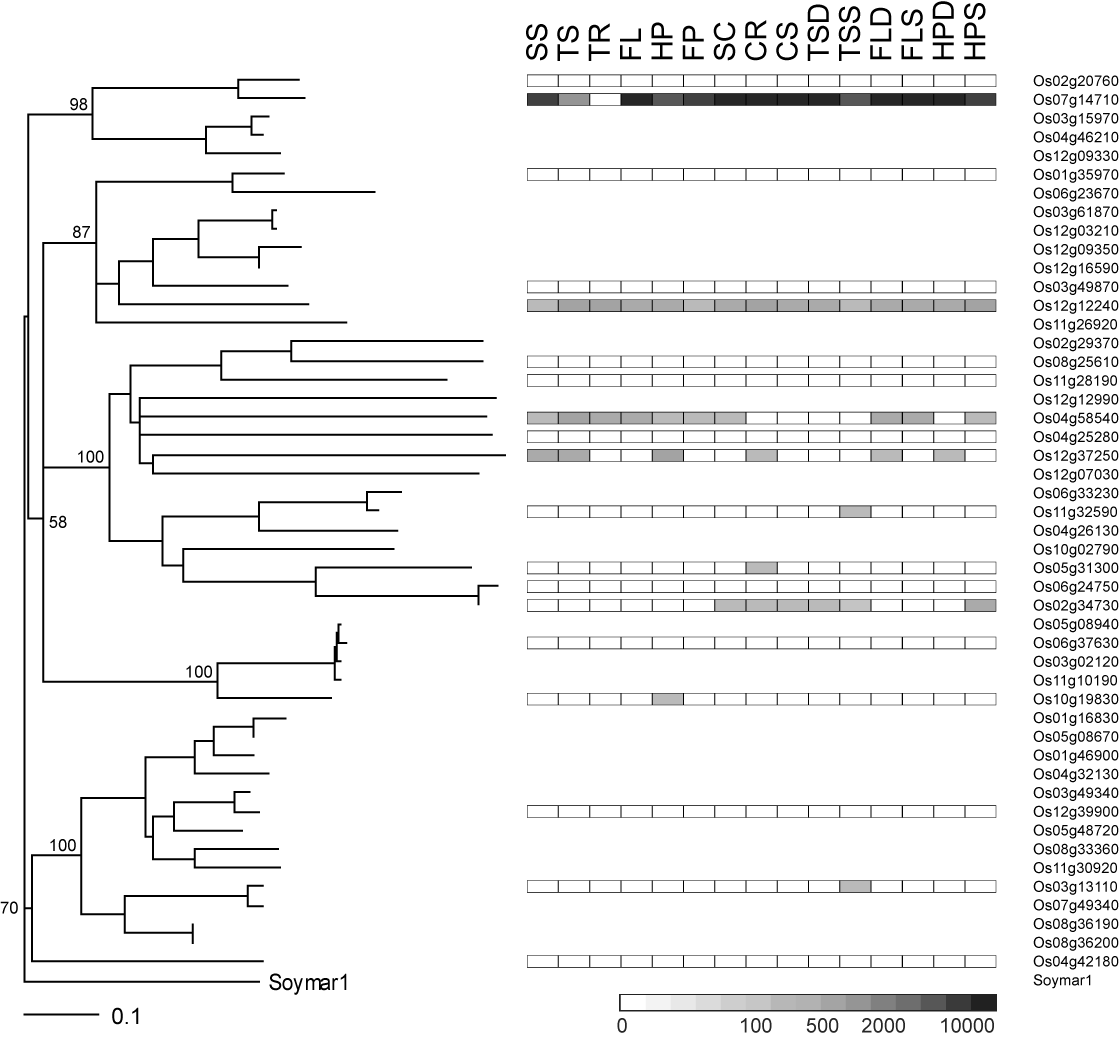

Supplement: Additional data file 4 — The phylogenetic tree was generated from a multiple alignment of conceptually translated sequences by using neighbor-joining methods and rooted with soybean Soymar1. Bootstrap values were calculated from 300 replicates. Sample numbers are identical to those in Table 2. Shades of gray indicate the magnitude of transcription signals, which are based on microarray hybridization signals without units. [file gb-2007-8-2-r28-S4.gif]
